# Madness

**DOI:** 10.3201/eid0809.020204

**Published:** 2002-09

**Authors:** Gerald N. Callahan

**Figure:**
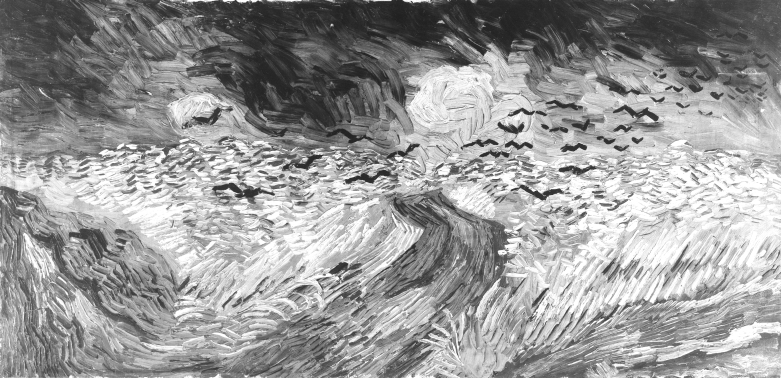
**Vincent van Gogh (1853-1890) “Crows in the Wheatfields” 1890**. Oil on canvas, 50.35 cm x 103 cm. Amsterdam, Van Gogh Museum (Vincent van Gogh Foundation)

 There are clouds in the painting, of course. Almost any one of us would have included those clouds, thick with electricity and rainwater. And there is the wheat field, smudged out like an empty palm, orange beneath the storm-stricken sun. Surely, many of us would have insisted on the wheat as well. Through the middle of the wheat, a rutted road slices to the horizon and disappears beneath the clouds. Even I, a scientist, would have included the road. A storm like that demands a road. Without the road, there is no hope at all. But then there are the crows—the one true hint of what had been and what was to come—fistfuls of them, flung into the swirls beneath the angry wet anvils. All that the painter had lost, irretrievably lost, he put inside those crows.

 Van Gogh died because of an instant (or a lifetime) during which the portrait of his life appeared worse than the portrait of his death. Died because his pictures filled up with crows. We call that a behavioral disorder because we imagine healthy people don’t see the crows, healthy people don’t choose death over life. And we say that behavioral disorders are caused by “mental” diseases to distinguish them from “real” diseases—infections, tumors, broken bones, burst blood vessels, polio. Real diseases are diseases of the *body*. 

 We do that—razor medicine off at the neck—because people such as René Descartes and Pope Urban VIII contended that the human soul resides in the mind, and human disease resides in the body. Sometimes because of that contention, we believe people with mental diseases are less genuinely ill than people with somatic diseases. Sometimes we even believe that people with mental diseases and behavioral disorders suffer more from weakness of spirit and flaws of character than from genuine disease. Beneath our collective breath, we say that the crows are inside their heads, and having said that, we imagine that the crows are not real. 

 Uncle Henry had a habit of leaving his fly unzipped, completely unzipped, regardless of who might be around to notice. My mother, his sister, hated that. Henry’s underpants were usually urine-stained, his shirt tails hung out of the opening in his pants, and he had a propensity to yell, “shit” and to spit for no apparent reason. Mother hated that too. And because she imagined that Henry’s eccentric behavior was concrete evidence of his total disregard for others, especially her, mother raised up a little hatred for Henry himself. Over the years, that hatred blossomed inside her and bore seed. 

 I don’t think Henry ever noticed how much mother despised him. At times, I’d watch him look at her with his sea-blue eyes, and I’d see something back there in the hollows. Whatever it was, though, it wasn’t shame, resentment, anger, or even understanding. Henry’s been dead now for more than 30 years, but mother still gets angry whenever I mention his name. I don’t understand that. Henry, I imagine, tried mother’s patience at times. But I’m certain he never intended to haunt her for 3 decades after his death. In fact, I don’t think Henry intended much of anything, at least not toward the end.

 Mother, though, is still tied to Henry, years after he cut his ties with everything and was laid to rest. And if she could explain today why she so despises Henry, mother would tell you it is because he was dirty and crazy. What she wouldn’t tell you (but you might discern if you listened to her for a moment or two) is that had Henry cared to, he could have stopped being crazy, just as easily as he could have stopped being dirty. Filth and craziness were just his ways of getting to her and making her life difficult.

## Instructional Film—Scene I 

A bucolic panorama somewhere in, say, Nigeria. The sky is first-morning blue, and a breeze tinged with wood smoke is ruffling the tall grass. There are cattle in the grass. A few are grazing, but most are standing or lying down lazily swatting flies with their tails. At first, the cattle are the only animals we see. But as the camera zooms in, we notice that the grass where the cattle sit or graze is home to brown ants and a few land snails, which at first glance seem in danger of being eaten by the cattle. As we watch longer though, we see that both ants and snails stay low enough in the grass to evade the foraging ruminants—idyllic, mutualistic Nigeria. Fade to black.

## Instructional Film—Scene II

The camera’s eye reopens a few miles to the north. The sun has fallen beyond the horizon, and the breeze wrestling with the grass has cooled. Most of the cattle here are grazing. As the camera closes in, we notice something oddly different from Scene I. Below the cattle, many of the ants have worked their way up the shafts of grass and appear to be waiting for something. As we watch in cinematic magnification, a pink tongue, large as a python, wraps itself around several insect-encrusted blades of grass, and brown ants (lots of them) suddenly disappear behind a huge set of cud-scarred teeth. Again, fade to black.

Let’s consider, for a moment, what we have just witnessed. In Scene I, the ants are cautious, responsible, and sane. In Scene II, the ants are none of those things. We are tempted, briefly, to say that the ants in Scene II are mentally ill. But we don’t say that, because we don’t imagine that ants have minds—at least not like human minds—so by definition, ants cannot be “mentally” ill. True or not, our belief serves a purpose; it cuts away some of the gauze that surrounds behavioral diseases in humans. That’s useful because the ants in Scene II clearly *are* crazy, whether we are comfortable with the words or not. They have climbed where they know they shouldn’t have and remain there in reckless disregard of the danger. They have lost the ability to care for themselves and seem to no longer value life more highly than death. These ants are insane, deranged, imbalanced, nutso. Men and women who behave similarly line the lavatory-colored halls of our country’s mental institutions from Passaic to Seattle. 

 Still, we don’t look for history of child abuse in ants, discuss ant toilet training, or accuse ants of character flaws and laziness. And we don’t imagine that ants are crazy or that their problems are all in their heads because, after all, these are ants. But if “mentally ill” isn’t accurate, what should we call them? Surprisingly, the answer to that question has come, not from psychologists or psychiatrists but from microbiologists, specifically parasitologists. And tedious dissections, not discussions, showed the way. 

 In Scene II (the one with the self-destructive ants), there was another actor (one we couldn’t see) at work inside every character portrayed. Because it is a very small actor, it is perhaps understandable that it escaped our attention. Everyone in Scene II was infected with a microscopic, lance-shaped fluke, a trematode called *Dicrocoelium dendriticum*. 

*D. dendriticum* is a parasitic flatworm. Parasitism is one of the oldest and most venerated ways of life on this planet. Living things have evolved to parasitize nearly all other living things—plants, animals, or microorganisms. Parasites themselves can be parasitized by smaller, but equally devious, life forms. And the animals and plants that do the parasitizing are as varied as their hosts. All things that parasitize animals fall into two groups, protozoa and helminths. Protozoa are single-celled animals such as *Plasmodium falciparum,* which infects nearly one-third of the world’s population and causes malaria. Helminths are worms—round, flat, and tape. *D*. *dendriticum* is a flatworm (or fluke as flatworms are sometimes called).

 In spite of their amazing variations, parasites have one characteristic in common: they cannot reproduce themselves outside their hosts. Parasites have lost the ability to perform one or more vital functions usually related to collecting or digesting food. Inside an animal’s body, the host takes care of these functions for them; outside the hosts, the parasites often die. Many parasites have evolved complex life cycles that help meet this uniform need in ways only parasites understand. These cycles involve several hosts and stages of development and necessarily feed back on themselves, so parasites end up in the same hosts where they began the cycles, and the whole process starts over.

*D. dendriticum* is like that. In fact, among parasites, *D. dendriticum* has one of the more interesting life cycles. Life begins for this fluke in the bile ducts of grazing cattle. This is where the adult flukes lay their eggs. Bile is produced in the liver and is transported, via the bile duct, to the intestine, where the bile aids in the digestion of dietary fats. In infected cattle, when the bile moves from the liver to the intestine, it takes *D. dendriticum* eggs along with it. A short while later, the eggs (along with the cow’s or bull’s feces) find their way onto the grasses underfoot. There, snails with inexplicable tastes ingest the cattle’s feces and the parasite’s eggs. Inside the snails, the eggs of *D*. *dendriticum* hatch, pass through two sporocyst stages of their lives, transform into another life stage called cercaria, and migrate to the respiratory chambers of the snails. Inside their respiratory chambers, snails make slime balls to aid movement across the fields. When the slime balls are secreted onto the snails’ feet, so are the cercaria. As the snails make their way toward whatever it is that draws snails, the slime balls are left behind in the grass.

 Slime balls are ant food. When ants eat them, they also eat the cercaria of *D.* *dendriticum*. Inside the ants, *most* of the cercaria encyst in the walls of the abdomen, but one or two migrate to the head and encyst in the sub-esophageal ganglion, a part of the brain. Here the cercaria transform into another life stage called metacercaria. Unlike the metacercaria left behind in the abdomen, these never become infective. These metacercaria do something else. They drive their hosts mad.

 As evening approaches and the air temperature drops, ants infected with *D.* *dendriticum *do not return to the colony along with their fellow workers. Instead, the infected ants climb to the tops of surrounding grasses, clamp their mandibles into the grass blades, and remain there, immobile, until the morning sun warms them again. When that happens, the ants (at least those who survive) resume their normal behavior—until the following evening. 

 Temporary insanity. “Temporary” because it lasts only as long as the sun is down. “Insanity” because the timing of the ants’ indiscretions corresponds exactly to the feeding cycles of the grazing cattle who feed most vigorously during the late evenings and early mornings. But here, the grasses are filled up with mad beings that suffer not from poor toilet training or moral and spiritual turpitude but from an infectious disease. Parasitic madness. Madness with a past and a purpose. 

 Each night beneath the African moon, crazy ants perch atop the grasses of Nigeria and wait for the cracked molars of hungry cattle to end a mad ritual. When the madness is complete and the ants are finally eaten, *D*. *dendriticum* completes its complex life cycle, and the arduous trip from cow to ground to cow closes once more. Inside the cow, digestive juices strip ant from parasite, and while the scene fades to black, life begins again, minus a few crazy ants. 

## Elsewhere in Nature

In the jungles of South America, there is another dance between ants and parasites. The parasite is a mushroom. Beneath tropical canopies, spores of Cordyceps*,* a mushroom*, *are whipped about on warm equatorial breezes and spun between tree trunks and twisting vines until they land in the spiracles of black ants. Spiracles (holes in the tough exoskeleton of ants) allow ants to breathe. Cordyceps uses the spiracles to get beneath ants’ skin. Once inside the ants, the fungus attaches to the soft tissues and begins to raise a family. For a few days, everything is fine, but the fungus knows that it will need more than the ants can provide. Soon, deep within the ants, the fungus will achieve sexual maturity, and it will be time to sporulate. Once again the infected ants, driven by the new-found energy of sudden acrophilia, leave the relative safety of the earth, climb atop the grass, clamp their mandibles onto the tips of the green shoots, and hang there. The fungus then consumes the ants' brains and sprouts through the emptied skulls. Bathed in sunlight, once again, the fungus sporulates. At the grass tops, where the wind blows freely, the spores are quickly spread, sometimes for miles and always to other ants. The fungus has lifted itself from the primordial slime, gathered itself upon the wind, and set off, once more, for a new life. Other varieties of Cordyceps mushrooms parasitize and alter the behavior of caterpillars, mealybugs, and beetles. Fungal madness. Infectious insanity.

 And then there's the odd story of *Wolbachia. *Most of us, I think, believe that genetics and evolution pretty much predetermine how we will reproduce ourselves. It seems unlikely that we have a choice about whether we procreate by mating with members of the opposite sex (as humans do) or by occasionally splitting ourselves in two (as bacteria usually do). But it turns out that, even though an animal’s reproductive behavior may not be of his or her own choosing, the behavior may not be a matter of genetics, evolution, or physiology either. 

 Some time ago, entomologists studying wasps and wood lice (which most of us call sow bugs) noticed that some species of these insects reproduce parthenogenetically, that is, without males (in fact without mating) and produce only (or mostly) female offspring. The entomologists concluded that these wasps and wood lice had evolved this method of reproduction to gain some advantage beyond our current understanding of biology—not to mention pleasure. 

 The entomologists were wrong. This sort of sexual behavior in wasps and wood lice isn’t normal. It’s a disease, an infectious disease. Entomologists leapt to the wrong conclusion because of something they couldn’t see, something hidden inside the wasps and the wood lice. *Wolbachia* *pipientis, *a bacterium and obligate intracellular parasite, lives in the ovaries and testes of many insect species. As many as 16% of insects (some 2 to 5 million species) may be infected with one strain or another of *Wolbachia*. But the bacterium is only transmitted vertically (mother to offspring), so only female insects can transmit the infection. To limit the number of male offspring, *Wolbachia* has developed ways to manipulate its host’s sex life.

*Wolbachia *interferes with the production and function of hormones and changes infected male wood lice into female wood lice. In other insects*, *the bacterium induces a state of “cytoplasmic incompatibility” between males and females, which prevents males and females from any productive mating. And in some wasps, it has completely eliminated males from the species. For these wasps to survive, the females must resort to parthenogenesis, and under these conditions, they can produce only female offspring. *Wolbachia* isn’t the only bacterium capable of this sort of sex selection by elimination of males or alteration of male behavior. At least five other species of bacteria similarly eliminate males from insect species to accelerate bacterial transmission in the wombs of females.

 Males turned into females, entire species of sexually inept insects, species of insects in which males have disappeared altogether—all this aberrant behavior, all of these “behavioral disorders,” can be cured with antibiotics, eliminated completely by any of a number of drugs that destroy bacteria. Yes, but that’s ants and wood lice. Bugs. Mammals are a lot more complex than ants and wood lice, aren’t they? 

Rats are intermediate hosts for another parasite, a single-celled, protozoan called *Toxoplasma gondii. T. gondii* begins and ends its life cycle in domestic cats. The immune response that cats mount against this parasite forces the parasite into very tough cysts that are shed in cats’ feces. The cysts survive in soil for years waiting for an intermediate host, a rat, to eat them. Inside the rats, *T. gondii* resumes its life cycle. The ultimate goal of the parasite is to complete the cycle by returning to its primary host, the cat. Cats do not have a great fondness for dead animals. So *T. gondii* doesn’t kill its rodent host. But rats have a general fear of cats and avoid the scent (and urine) of cats at all costs, which slows the transmission of *T. gondii*. To overcome this bottleneck, the parasite has learned to make rats crazy. Rats infected with *T. gondii *show no fear of cat urine, not because they have no sense of smell, but because some of them develop an attraction—an often fatal attraction—to cat urine. They go mad and seem to invite their own deaths. Again, no dysfunctional family, birth defect, or blow to the head made these animals crazy. This madness has an infectious cause.

*T. gondii *infects people too. Gardening in cyst-infested soil, handling infected meat, or emptying litter boxes used by infected cats can result in infection. In fact, nearly half the people in this world have *T. gondii* cysts in the brain. *T. gondii* has never figured out a way to make humans palatable to cats, but that doesn’t mean people are unaffected by the parasite. In psychological tests, women with *T. gondii* cysts in the brain were more outgoing and warm-hearted than uninfected controls, and men infected with the parasite were more jealous and suspicious than uninfected men—behavior with a twist, a protozoan twist.

 No one knows what drove Vincent van Gogh to take his own life; depression was part of it, certainly. But depression alone seems an insufficient explanation. In the year before his death, the painter enthusiastically brought Paul Gaugin to join him in Arles. Less than 2 months later, van Gogh attacked his guest with a straight razor; then in remorse, cut off his own ear and offered it to a local prostitute. In the same year, van Gogh painted “Sunflowers,” a celebration of yellows and browns, and “Starry Night,” a tableau of haunting blues and swirling stars—a portrait of the abyss itself. Within a few months of one another, he painted the inviting “Bedroom at Arles” and a brutal self-portrait that leaves the viewer nowhere else to look. Later that same year, he painted “Starlight over the Rhine” and “Wheatfield with Crows.”

 Those dramatic swings from euphoria to abject despair suggest bipolar disorder, manic-depression. At the height of his mania, van Gogh painted “Sunflowers.” In the pit of depression, he painted his last work, “Wheatfield with Crows.” Bipolar disorder is a “behavioral” disorder, a mental disease of unknown cause. Many of us imagine that in the bipolar patient, the crows are all inside the mind. We may be wrong. Perhaps it was all in van Gogh’s head, all in his mind. But even if we are unwilling to change our thoughts about what “mind” means, we may have to change our thoughts about what “all” means.

 Rats, tree shrews, and monkeys (mammals like us—some much like us) infected with Borna disease virus behave much like humans with bipolar disorder. These animals exhibit periods of apparent mania and periods of obvious depression. They are more anxious, less sexually active, less interested in food, and have a greater desire for salt—just like manic-depressive humans. All because of a virus, another obligate intracellular parasite. And because of that virus, infected animals—only infected animals—have abnormalities that mimic a devastating behavioral disorder of humans.

 Humans also become infected with Borna disease virus, and those infected appear more susceptible to certain “behavioral” disorders. At autopsy, nucleic acid from Borna disease virus has been found in the brain of a disproportionately high number of patients with bipolar disorder, severe depression, and schizophrenia. Viral madness?

 Obsessive-compulsive disorder (OCD) manifests as the inability to resist or stop continuous abnormal thoughts; fears; or ritualistic, repetitive, and involuntary behavior. People with OCD may not be able to stop washing their hands, stop hoarding things, stop checking if they’ve turned the stove off, or stop driving around the block to look for accidents and their victims. OCD is a mental disorder, experts tell us, a behavioral anomaly. 

 Unexpectedly, though, a substantial number of children with obsessive-compulsive disorders have first signs of illness a few weeks after streptococcal infection—strep throat. Streptococci are infectious bacteria, the same bacteria that cause scarlet fever, rheumatic fever, glomerulonephritis, and other diseases. Apparently after strep infections, our immune systems may mistake our own cells for our enemies. In some patients with OCD, the immune system’s enemy appears to be part of the brain. These patients’ immune systems produce antibodies that attack the cells of the brain, and almost overnight, the “craziness” that we call OCD can develop. Antibiotics, which kill streptococci, often relieve the symptoms.

 Here an infectious disease is amplified somehow by a person’s own immune system, and abruptly someone we once called “sane” can’t get it out of her head that she is going to harm her own children, can’t stop counting the silverware, can’t stop scrubbing her hands, can’t stop thinking about murdering her husband. 

 And then there’s Uncle Henry, the one who disgusted mother. His cursing, personal habits, and appearance were odd, I’ll admit. All of us, I suppose, imagined he was a little crazy. Mother imagined it more than most. And you remember, she resented his craziness most of all. As it turned out, though, there was more to Henry than met our eyes. Henry had syphilis, an infectious disease that’s been around since at least the 16th century when it was named the “great pox.” A bacterium, a spirochete called *Treponema pallidium*, causes syphilis. *T.* *pallidium* is transmitted through small breaks in the skin that occur during sexual intercourse. Within a few months, the bacteria spread from the point of entry to the lymphatics, the joints, and the skin. And, in 10% of people who go long enough without treatment, bacteria spread to the brain and spinal chord. Once inside the brain, the spirochetes cause some paralysis and progressive dementia. Apparently, Henry was among that 10%.

 By 1941, when Alexander Fleming finally got around to developing penicillin to the point of clinical application, it was too late for Henry. Before then, Paul Ehrlich and others used “Salvorsan,” a compound that contained, among other things, arsenic. Sometimes it worked, and sometimes it didn’t. But often the infected understood so little about the disease that if they sought treatment at all, it was usually too late. And even now, antibiotics are of little use after the spirochete is in the brain. Then, there is only an unstoppable, progressive, “mental” disease. So for many like Henry, uneducated about the dangers of casual sex and infected before Fleming’s penicillin, there was only a final madness. 

 In spite of that, mother still resents Henry, and, I’m sure, the nature of his infection. I doubt that mother ever connected the two, ever imagined that the fire beneath the boiler of Henry’s craziness was being stoked by bacteria. She never mentioned Henry’s disease until I was much older. Old enough, I guess, that she thought I might understand, even though *she *never did. And she never forgave him, never believed that all of it wasn’t Henry’s fault—the disease, the craziness, the indiscretions. She discounted the disease and laid the blame squarely on Henry. She hated him for that. She hated him too, I’m sure, for the way his craziness freed him from all responsibility and left her to bear his shame.

 Now, mother has Alzheimer’s, another disease with no known cause. Some believe an infectious agent is involved—maybe so, maybe not. As the disease progresses, a protein called amyloid is deposited in mother’s brain, and parts of the brain are slowly disappearing. At autopsy, brains from Alzheimer’s patients often resemble wispy growths of pale coral with deep fissures and frail fins. Everything I believed was mother is slowly yielding to the disease. Her poverty is nearly complete, the disease nearly crystalline, and the craziness fulminant. Now, she rarely recognizes my father (her husband of more than 60 years) or any of her four children. Long-term memory seems more resistant. The mention of Uncle Henry still causes her to twist her lip into a sneer and curse him for his craziness. 

 We are told that van Gogh killed himself because he was depressed or crazy or both. But who knows? Who knows what truly tortured this man or what more (if anything) was beneath the lurid oils and feverish brush strokes? Regardless, for at least one moment, the picture of his death seemed less terrifying to him than the picture of his life. And though he may have tried to prevent it, that dark picture finally crawled out of the end of his brush and onto the canvas. It was all suddenly there in the crows. He saw that, and when he was finished, he stared long and hard at what he had done, covered the painting, and left the studio. A week or so later, he shot himself, perhaps repeatedly. Two days after that, he died.

**Sources**

1. Zimmer C. Parasites make scaredy-rats foolhardy. Science 2000;289:525b.

2. Hitalski CG, Lewis AJ, Lipkin WI. Borna disease. Emerg Infect Dis 1997;3:129.

3. Travis J. Undesirable sex partner: bacteria manipulate reproduction of insects and other species. Science News 1996;150:228. 

4. Swedo SE, Leonard HL, Garvey M, Mittleman B, Allen AJ, Perlmutter S, et al. Pediatric autoimmune neuropsychiatric disorders associate with streptococcal infections: the first 50 cases*.* Am J Psychiatry 1998;155:264–71.

5. Weschler L. Mr. Wilson's cabinet of wonder: pronged ants, horned humans, mice on toast and other* m*arvels. New York: Vintage Books; 1996.

